# Draft genome sequences of *Streptomyces* strains from the H62 and H72 from the Atacama Desert, Chile

**DOI:** 10.1128/mra.01119-25

**Published:** 2026-03-23

**Authors:** Weijie Zhou, Jenileima Kshetrimayum Devi, Nick Allenby

**Affiliations:** 1 John Dawson Drug Discovery Centre, University of Sunderland, Sunderland, United Kingdom; DOE Joint Genome Institute, Berkeley, California, USA

**Keywords:** *Streptomyces*, genome sequencing, biosynthetic gene clusters, Oxford Nanopore Technologies, antimicrobial, natural products

## Abstract

We report draft genome sequences of two *Streptomyces* strains isolated from Atacama Desert soil in Chile. Strains H62 and H72 have genome sizes of 7.9 and 8.0 Mbp with GC contents of 72.4% and 72.3%, respectively, and harbor multiple biosynthetic gene clusters.

## ANNOUNCEMENT

The Atacama Desert is among the harshest terrestrial environments, where microbial life has adapted to extreme aridity, UV radiation, and nutrient limitation (1, 2). Previous genomic surveys of Atacama isolates revealed enrichment of biosynthetic gene clusters (BGCs), underscoring their potential as reservoirs of new antimicrobial compounds (3–5). Therefore, we analyzed bacterial isolates from Atacama Desert soil samples to assess their antimicrobial potential.

Soil sample ALMA2 was collected aseptically from subsurface soil (30 cm depth) in the Atacama Desert, Chile (23°04′39″S, 67°57′43″W; 3,018 m above sea level) on 26 October 2012. Sampling implements were sterilized in the field with ethanol, and material was placed into sterile polycarbonate bottles. Samples were transported to the UK and stored at 4°C until processing (6). *Streptomyces* strains H62 and H72 were isolated at Newcastle University from soil sample ALMA2 by dilution plating on yeast extract-malt extract agar at 28°C (7–9). The isolates were later transferred to the University of Sunderland, where they were maintained as glycerol stocks at −80°C for downstream sequencing and analysis (7).

Genomic DNA was extracted from *Streptomyces* strains H62 and H72, which were cultivated on ISP2 agar (7) at 30°C for 48 h. Mycelial biomass was collected by gently scraping the surface of the agar plates with a sterile loop and suspended in DNA/RNA Shield solution (Zymo Research, USA) for preservation. The harvested cells were stored at −20°C until further processing. High-molecular-weight genomic DNA was isolated using the Quick-DNA HMW MagBead kit (Zymo Research, USA), following the manufacturer’s protocol. DNA purity was measured by Qubit 3.0 fluorometer (Invitrogen, USA), yielding concentrations of 33.0 ng/μL for strain H62 and 20.6 ng/μL for strain H72. The integrity of the extracted DNA was verified by agarose gel electrophoresis (10). Library preparation was carried out using the Ligation sequencing V14 kit with native barcoding expansion (SQK-NBD114.24; Oxford Nanopore Technologies [ONT], UK), following the manufacturer’s protocol (11). Then, 400 ng DNA from each strain was used in the end-repair reaction. Equimolar pools of barcoded samples were prepared, sequencing adapters were ligated, and final libraries were sequenced on a MinION Flow Cell (ONT, UK). Basecalling was performed in real time using Dorado v0.7.1 via the MINKNOW platform (Oxford Nanopore Technologies, UK). Sequencing statistics are summarized in Table 1. Quality assessment with NanoPlot v1.44.1 (12) showed that >99% of reads were above Q10 for both strains. Given the high read quality and sufficient coverage, raw reads were directly used for genome assembly using Flye v2.9.5 (13) without additional filtering. The resulting assemblies were polished with Medaka v1.11.3 (ONT) (14). Polished assemblies were then annotated with Bakta v1.10.1 (15), and biosynthetic gene clusters were predicted with antiSMASH v7.1.0 (16). Genome completeness was assessed using CheckM v1.2.3 (17), indicating >95% completeness and <5% contamination for both strains. Default parameters were used for all software except where otherwise noted.

**TABLE 1:** Draft genome sequences of two *Streptomyces* strains isolated from soil samples in the Atacama Desert, Chile

Strain	H62	H72
Species	*Streptomyces*	*Streptomyces*
Sequencing statistics		
Total reads	108,174	106,020
Sequencing yield (Mb)	694.2	681.5
Mean read length (bp)	6,418	6,428
Median read length (bp)	2,853	2,570
Read N50 (bp)	14,112	15,237
Mean read quality (Q)	15.6	15.7
Reads >Q10 (%)	99.3	99.4
Strain assembly statistics		
Genome size (bp)	7,881,201	8,006,860
Contigs	3	3
Assembly N50 (bp)	7,685,310	7,688,050
GC content (%)	72.36	72.29
Coverage (×)	86×	84×
Completeness (%)	100	100
Contamination (%)	0.57	0.57
Annotation statistics		
CDS	6,843	6,996
tRNA	78	80
rRNA	18	18

**Fig 1:**
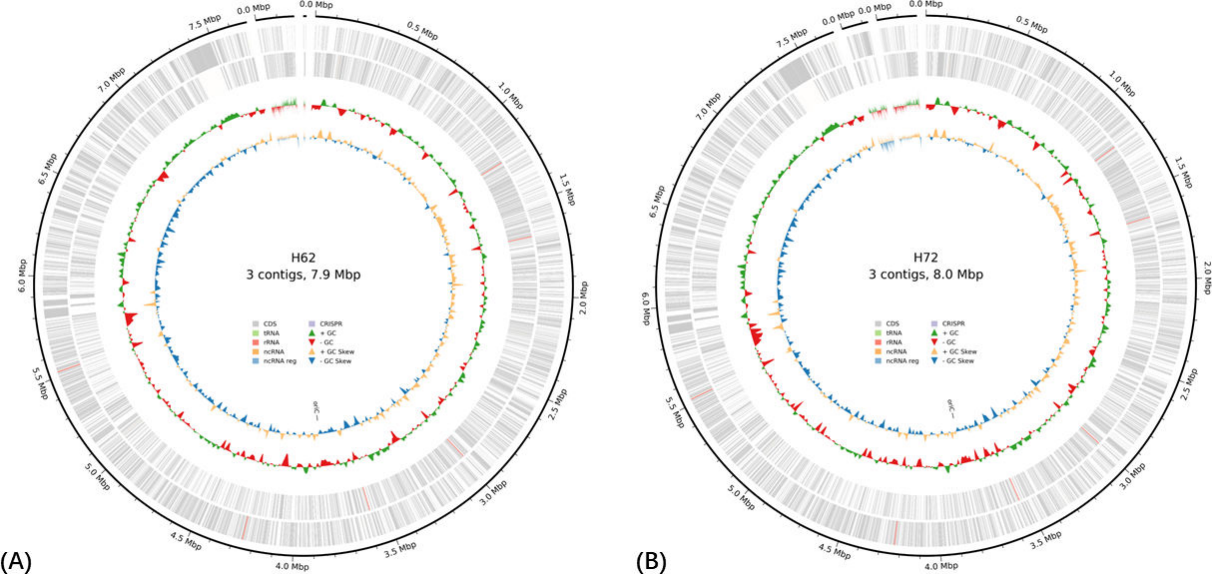
Circular representation of the annotated genome of *Streptomyces* strains H62 (**A**) and H72 (**B**).

Assembly and annotation statistics for strains H62 and H72 are presented in Table 1, while circular representations of the genome are shown in Fig. 1. Strains H62 and H72 share 99.4% ANI, based on the results of FastANI v1.34 (18) consistent with strain-level divergence. Genome mining identified 28 and 29 BGCs, respectively (antiSMASH v7.1.0, --genefinding-tool prodigal, --taxon bacteria, --cc-mibig, --cb-general, --cb-knownclusters, and --cb-subclusters), with the additional cluster in H72 annotated as lanthipeptide-class-i located on contig 3, suggesting a minor difference in biosynthetic capacity. These findings highlight the potential of *Streptomyces sp*. strains H62 and H72 as a source of new antimicrobial metabolites.

## Data Availability

Raw sequencing reads have been deposited in the NCBI Sequence Read Archive under accession numbers SRR35636520 (strain H62) and SRR35636519 (strain H72), associated with BioProject PRJNA1333581 and BioSample accessions SAMN51802655 and SAMN51802656, respectively. Genome assembly accessions are JBRILH000000000 (strain H62) and JBRKLD000000000 (strain H72).
